# Metabolomics combined with transcriptomics analysis on ginsenosides accumulation in root of American ginseng plants under foliar applications of brassinolide

**DOI:** 10.3389/fpls.2026.1798852

**Published:** 2026-07-03

**Authors:** Fangzhou Zhao, Huili Tang, Debao Huang, Jianhua Zhang, Jiaxin Zhang, Hongxia Yu, Feiyan Ju, Xin Wang, Hongqing Xie, Li Ren, Yujuan Zhang, Yuanyuan Duan, Shaopeng Yi, Jiaohong Zhang, Jinlong Han, Yun Zhou, Chenggang Shan

**Affiliations:** 1 Institute of Industrial Crops, Shandong Academy of Agricultural Sciences, Jinan, China; 2 Department of Horticultural Science, North Carolina State University, Raleigh, NC, United States; 3 College of Agronomy, Qingdao Agricultural University, Qingdao, China; 4 Weihai (Wendeng) Authentic Ginseng Industry Development Co., Ltd, Weihai, China

**Keywords:** American ginseng, brassinolide, ginsenosides, metabolomics, transcriptomics, triterpenoid biosynthesis

## Abstract

*Panax quinquefolius* L. (American ginseng) is a medicinally important crop with high pharmacological value, but its commercial application is restricted by low ginsenoside yields and insufficient bioavailability. In this study, we performed integrated metabolomic and transcriptomic analyses to explore the regulatory effects of foliar-applied brassinolide (BL) on American ginseng. BL exhibited a dose-dependent effect on the coordination between plant growth and ginsenoside accumulation. Low-concentration BL promoted plant growth and induced the accumulation of five rare ginsenosides (ginsenoside F5, pseudoginsenoside Rt3, majoroside R2, ginsenoside F3, majoroside R1) in roots by 2–21-fold. High-concentration BL displayed no growth-promoting effect but significantly enhanced the accumulation of the same ginsenosides by 11–75-fold. Transcriptomic analysis identified 34 differentially expressed genes (DEGs) involved in the ginsenoside biosynthesis pathway. Weighted gene co-expression network analysis (WGCNA) revealed 16 CYP450/UGT genes and 17 transcription factors as key regulators mediating BL-induced rare ginsenoside biosynthesis. Multi-omics integration further showed that four β-amyrin synthase genes (*PQ0G073990, PQ0G318090, PQ0G569630, novel.5640*) and two CYP450 genes (*novel.16917, PQ0G686990*) were co-enriched with oleanane-type saponin Ro O-pentoside and downregulated by high-concentration BL. These results illustrate the dual function of BL in balancing plant growth and secondary metabolism, providing candidate molecular targets for metabolic engineering and sustainable cultivation practices aimed at improving rare ginsenoside production in American ginseng.

## 1 Introduction

*Panax quinquefolius* L. (American ginseng) is a globally renowned medicinal plant valued for its dual use as both food and medicine. It is values for its diverse array of bioactive compounds and pharmacological effects, including hypoglycemic, cardiovascular protective, and antitumor activities (Assinewe et al., 2003; Szczuka et al., 2019). Ginsenosides, the primary bioactive components of American ginseng, serve as key quality markers for this herb. However, traditional cultivation methods faces several challenges, including genetic heterogeneity, extended growth cycles, geographical constraints, and low ginsenoside yields, which limit the ability of this herb to meet growing pharmaceutical demands (Liang et al., 2019).

More than 100 ginsenosides have been identified in *P. quinquefolius*, classified into four main types based on their triterpenoid skeletons: protopanaxadiol (PPD), protopanaxatriol (PPT), ocotillol (OCT), and oleanane (OA) types (Yuan et al., 2010; Hou et al., 2021). Among these, PPD- and PPT-type ginsenosides (e.g., Rb1, Rg1) predominate, accounting for over 90% of the total ginsenoside content. However, the large molecular size and poor membrane permeability significantly limit their oral bioavailability in humans (Yang et al., 2022). In contrast, rare ginsenosides - although pharmacologically potent - are present in low abundance in planta, necessitating innovative strategies to enhancing their production. One promising approach involves reconstructing the biosynthetic pathways in engineered microorganisms, which requires the identification of key enzymes involved in ginsenosides metabolism (Ren et al., 2022; Li et al., 2024a).

Ginsenoside biosynthesis is primarily mediated via the mevalonic acid (MVA) pathway, initiated by squalene epoxidation to (S)-2,3-oxidosqualene, followed by cyclization catalyzed by oxidosqualene cyclases (OSCs) and subsequent modification by cytochrome P450 monooxygenases (CYP450) and UDP-glycosyltransferases (UGT) (Kim et al., 2015a). While the genes involved in the upstream MVA pathway are well-characterized, the downstream enzymes (e.g., OSC, CYP450, UGT) remain poorly understood, thereby limiting the progress of metabolic engineering efforts (Mohanan et al., 2023; Tian et al., 2024).

Brassinosteroids (BRs), a class of polyhydroxysteroid hormones, play crucial roles in regulating plant growth, secondary metabolism, and responses to environmental stress (Han et al., 2023). In *P. quinquefolius*, BRs have been implicated in the regulation of ginsenoside biosynthesis, although the underlying mechanisms remain largely unclear (Zheng et al., 2023). Previous studies highlight the multifaceted functions of BRs in growth regulation: in *Malus hupehensis*, exogenous BRs promoted growth by activating the auxin pathway (Mao et al., 2017), whereas in *P. ginseng*, the BR analog brassinolide (BL) inhibited xylem vessel formation during secondary growth (Hwang et al., 2020). In several medicinal plants, BRs have also been shown to influence the production of secondary metabolite. For example, foliar application of BL enhanced alkaloid and flavonoid accumulation in *Pinellia ternata* (Guo et al., 2022), and modulated diosgenin homeostasis in *Dioscorea zingiberensis* through cycloartenol synthase (CAS) and CYP90 gene regulation (Li et al., 2024b). Despite these findings, the effects of foliar-applied BRs on ginsenoside accumulation and gene expression in *P. quinquefolius* roots remain unexplored.

Leveraging omics technologies-transcriptomics and metabolomics-provides a powerful approach for dissecting complex metabolic networks. In this study, we employed an integrated multi-omics approach to investigate the effects of BL treatment on *P. quinquefolius* growth and ginsenoside biosynthesis. Specifically, our objectives were to: 1) assess growth responses under varying BL concentrations; 2) identify key metabolic changes and regulatory genes associated with ginsenoside biosynthesis; and 3) elucidate the underlying mechanisms to inform strategies for optimizing BR-mediated ginsenoside accumulation. This study addresses a critical knowledge gap and advances our understanding of BR-regulated secondary metabolism, offering valuable insights for sustainable cultivation and biotechnological improvement of American ginseng.

## 2 Materials and methods

### 2.1 Plant materials and growth conditions

Seeds of American ginseng cultivar ‘LYS1’ were surface sterilized by soaking in 2% (w/v) sodium hypochlorite for 30 minutes, followed by three rinses with sterile deionized water. The sterilized seeds were sown in a soil-sand mixture (2:1, v/v) that had been autoclaved at 121 °C and 0.10 MPa for 2 hours, and placed in 17 cm × 15 cm pots. Plants were grown in a controlled environment chamber under the following conditions, 25 ± 2 °C, 70 ± 5% relative humidity, 16/8 h (light/dark) photoperiod, and photosynthetic photon flux density of 100 μmol m^-^² s^-^¹. Immediately after establishment, the seedlings were transplanted to shaded farmland under natural long−day conditions in Wendeng District, Weihai City, Shandong Province, China. Seedlings were planted individually with a row spacing of 15 cm and a plant spacing of 10 cm.

### 2.2 Brassinolide treatments

Two-year-old American ginseng plants at the leaf-expansion stage were foliar-sprayed using a 20 L electric backpack sprayer until runoff to ensure uniform coverage. Plants were divided into two groups, with three biological replicates per group: (1) the control group, treated with water containing 0.1% Tween 20; (2) the treatment group, sprayed with brassinolide (BL) solutions at six concentration solutions. These solutions were prepared by diluting the BL solution (0.1 mg/mL) purchased from Qingdao Hansheng Biotechnology Co., Ltd. (Qingdao, China) to 15000-fold (S1), 12000-fold (S2), 8000-fold (S3), 6000-fold (S4), 4000-fold (S5), and 2000-fold (S6), respectively, with each dilution also containing 0.1% Tween 20. Treatments were performed three times at 7-day intervals. Growth parameters, including plant height, stem diameter, leaf area and root samples, were collected on days 0 and 21 post-treatment. The growth rate is defined as the ratio of the difference in growth parameters measured before and after treatment to the pre-treatment growth parameters.

### 2.3 Morphological and ginsenoside analyses

Root samples were divided into two subsets: one oven-dried at 60 °C for ginsenoside quantification, while the other flash-frozen in liquid nitrogen and stored at −80 °C for subsequent omics profiling. Six major ginsenosides (Rg1, Rd, Rb1, Rb2, Rb3, Re) were quantified using high-performance liquid chromatography (HPLC) following the method described by Zhang et al. (2024). Briefly, chromatographic separation was performed on an Agilent ZORBAX SB-C_18_ column using a gradient elution with a mobile phase composed of solvent A (100% acetonitrile) and solvent B 0.1% (0.1% aqueous solution of phosphoric acid). The gradient program was applied at 35 °C as follows: 0–14 min, 20–21% A; 14–20 min, 21–22% A; 20–26 min, 22–23% A; 26–38 min, 23–25% A; 38–42 min, 25–28% A; 42–60 min, 28–30% A; 60–74 min, 30–35% A; 74–86 min, 35–55% A; and 86–98 min, 55–75% A. The injection volume was 10 μL, with a constant flow rate of 1.0 mL min^−1^. UV-spectrometry detection was conducted at a wavelength of 203 nm. Quantification was achieved based on the standard curves established from multi-point concentration gradients (Zhang et al., 2024). Six gradient concentrations of mixed reference standard solutions were prepared in the study, and standard curves were plotted with concentration as the abscissa and peak area as the ordinate to obtain the linear regression equations. The external standards of authentic ginsenoside were obtained from Shanghai Yuanye Bio-Technology Co., Ltd. (Shanghai, China).

### 2.4 Metabolomic analysis

Metabolomic analysis was performed by Metware Biotechnology (Wuhan, China). Root samples of American ginseng plants in the control group and treatment groups treated with 15,000-fold (S1) and 8,000-fold (S4), which were stored at −80 °C, were freeze-dried using a Scientz-100F freeze dryer (Ningbo, China). The freeze-dried root materials were ground using an grinder (MM 400, Retsch) at 30 Hz for 1.5 min.

For metabolomic analysis, a 50 mg aliquot of each sample was extracted with 1.2 mL pre-cooled 70% methanol containing internal standards. The extracts were vortexed for 30 seconds every 30 minutes over 6 cycles, then centrifugated at12000 rpm for 3 minutes. The resulting supernatants were filtered through 0.22 μm membranes and subjected to UPLC-MS/MS analysis using a Shim-pack UFLC SHIMADZU CBM30A system coupled with an Applied Biosystems 4500 Q TRAP Mass spectrometer. The ESI source parameters were set as follows: source temperature 500 °C; ion spray voltage (IS) 5500 V (positive ion mode)/-4500 V (negative ion mode); ion source gas I (GSI), gas II (GSII), curtain gas (CUR) were set at 50, 60, and 25 psi, respectively; the collision-activated dissociation (CAD) was high. QQQ scans were acquired as MRM experiments with collision gas (nitrogen) set to medium. DP (declustering potential) and CE (collision energy) for individual MRM transitions were done with further DP and CE optimization. A specific set of MRM transitions were monitored for each period according to the metabolites eluted within this period. Metabolites were quantified via multiple reaction monitoring (MRM), and data were processed with Analyst 1.6.3 software. Differentially accumulated metabolites (DAMs) were identified based on the thresholds of |log_2_(fold change)| ≥ 1 and variable importance in projection (VIP) ≥ 1, as determined using the MetWareCloud platform (cloud.metware.cn), a free online platform for data analysis. KEGG pathway enrichment analysis was performed with reference to the KEGG database.

### 2.5 Transcriptome sequencing and analysis

The total RNA was extracted using the CTAB-PBIOZOL method (Mu et al., 2017). The concentration of the extracted RNA was quantified using a Qubit 4.0 fluorometer, and its integrity was determined with the Qsep400 system (Bioptic, Taiwan). mRNA was isolated from total RNA using magnetic beads conjugated with oligo (dT) primers, following the protocol of the Dynabeads™ mRNA purification Kit (Invitrogen, USA). The purified mRNA was reverse-transcribed into single-stranded cDNA using PrimeScript RT Reagent Kit with gDNA Eraser (Takara Bio Inc., Japan). Double-stranded cDNAs were synthesized using dNTPs, high-fidelity DNA polymerase, and the appropriate buffer. Then, the resulting cDNAs were then end-repaired, A-tailed and ligated with sequencing adaptors. The fragments ranging from 250–350 bp cDNA were selected using AMPure XP beads to constructed Libraries (Beckman Coulter, USA). Sequencing was carried out on the Illumina HiSeq platform. Raw reads were processed to remove ambiguous bases (multiple “N”), adapter sequences, and low-quality bases (Q ≤ 20), yielding high-quality clean reads. These clean reads were subsequently aligned to the reference genome of American ginseng (Accession No. GWHBEIR00000000.1) (Wang et al., 2022), obtained from the China National Center for Bioinformation (https://ngdc.cncb.ac.cn/gwh/Assembly/22237/show) using HISAT2 (v.2.0.1) (Kim et al., 2015b). Transcript abundance of each gene was quantified in fragments per kilobase of transcript per million mapped reads (FPKM) using FeatureCounts v.1.5.0 (Liao et al., 2014). The differentially expressed genes (DEGs) among samples were identified using DESeq2 software (Love et al., 2014; Varet et al., 2016) according to the raw counts with |log2FC| ≥ 1 and false discovery rate (FDR) < 0.05 as thresholds. Functional annotation was performed using public databases, including the NCBI non-redundant (NR) nucleotide sequences database, the Protein Family (Pfam) database, Swiss-Prot database, Gene Ontology (GO) database, Klusters of Orthologous Groups (KOG) database, and Kyoto Encyclopedia of Genes and Genomes (KEGG) database.

### 2.6 Weighted gene co-expression network analysis and co-expression network

WGCNA was performed to construct co-expression network modules that associate gene expression pattern with metabolite profiles. Correlations analysis between the gene expression network modules and ginsenosides fractions was carried out to identify the specificity module-trait associations, using the MetwareCloud platform. The top 20 hub genes within the key models were identified based on their correlation with other genes in the network. The co-expression networks were visualized using the Metware Cloud Platform.

### 2.7 Quantitative real-time PCR

To validate the transcriptome sequencing (RNA-Seq) results, quantitative real-time PCR (qPCR) was performed on a CFX Duet Real-Time PCR System (Bio-Rad Laboratories, Inc., Hercules, CA, USA). The reactions were carried out using ChamQ SYBR qPCR Master Mix (Vazyme, Nanjing, China) following this protocol: initial denaturation at 95°C for 2 min; 39 cycles of denaturation at 95°C for 5 s and annealing at the pre-determined optimal temperature for 30 s and a final incubation at 65°C for 5 s. A melting curve analysis (ranging from 65°C to 95°Cwith 0.5°C increments per step) was conducted post-amplification to confirm the specificity of primer binding and avoid non-specific amplification products. For normalization, PqActin served as the internal control (Kochan et al., 2019). The relative quantification of gene expression was calculated using the 2^−ΔΔCT^ method. Each experimental condition included three biological replicates, each analyzed in triplicate to ensure data reliability. The gene-specific primer sequences used in this study are provided in **Supplementary Table 10**.

### 2.8 Statistical analysis

Data were analyzed using SPSS Statistics software (version 19.0, SPSS, Chicago, USA) through analysis of variance (analysis of variance). Mean differences were evaluated using the least significant difference (LSD) test at a significance level of *p*-value < 0.05. Figures were generated using Origin 2021 and Adobe Illustrator 2021.

## 3 Results

### 3.1 Changes in plant growth and ginsenoside content in roots of American ginseng under foliar application of brassinolide

In this study, American ginseng leaves were treated with brassinolide (BL) solutions, which were prepared by diluting the BL stock solution to six different concentrations: 15000-fold (S1), 12000-fold (S2), 8000-fold (S3), 6000-fold (S4), 4000-fold (S5), and 2000-fold (S6). Plant growth parameters and ginsenoside contents were determined at 21 days post-treatment.

**Figure 1:**
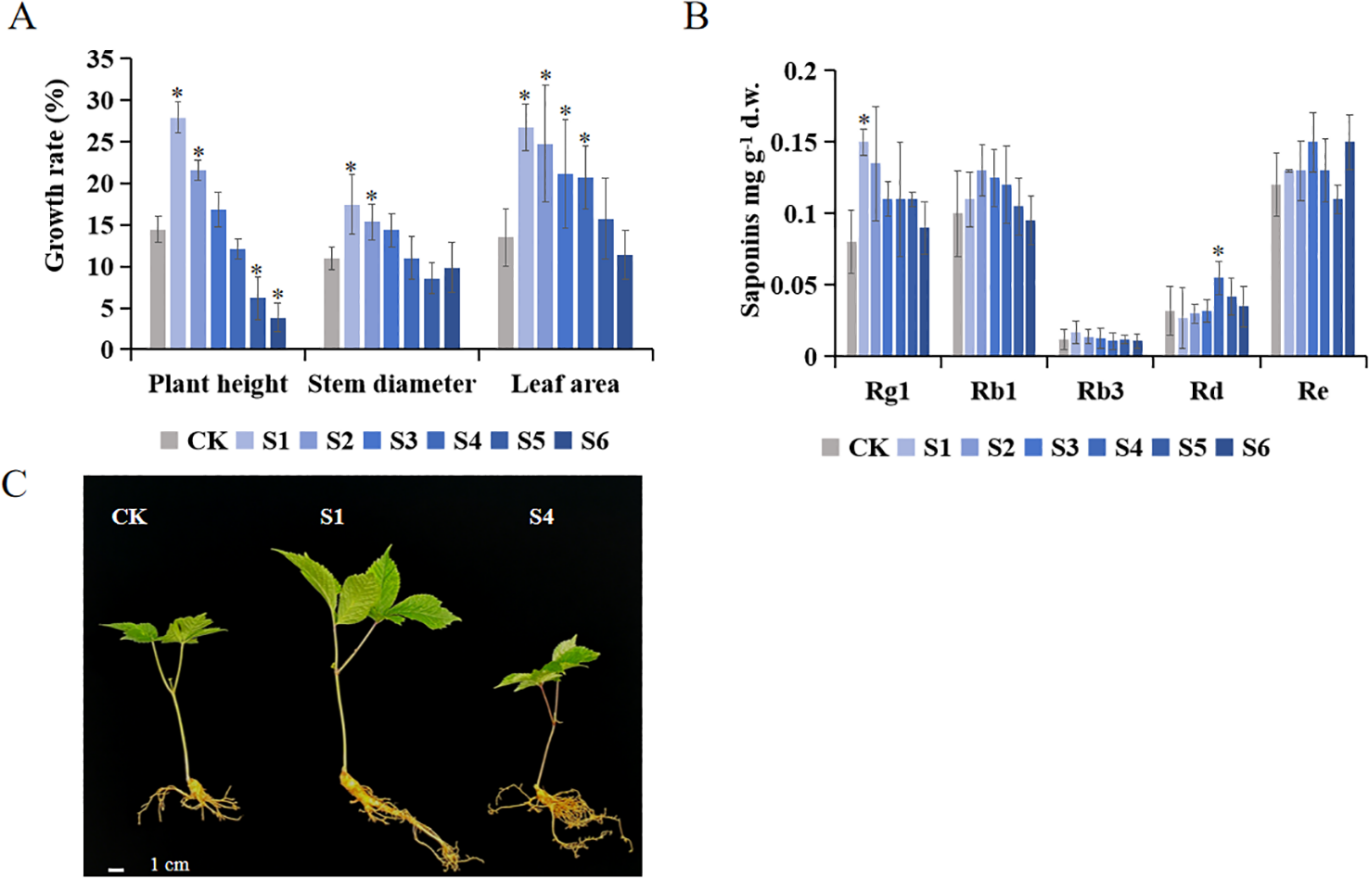
Effects of foliar application of brassinolide at different concentrations on plant growth and ginsenoside content in American ginseng roots. **(A)** Plant height, stem diameter, and leaf area. S1–S6 indicate six different concentrations of BL solution diluted from the BL stock: 15,000-fold (S1), 12,000-fold (S2), 8,000-fold (S3), 6,000-fold (S4), 4,000-fold (S5), and 2,000-fold (S6), with CK indicating the control. **(B)** Contents of ginsenosides Rg1, Rb1, Rb3, Rd, and Re. **(C)** Typical images of the seedlings. Column bars represent the mean of three biological replicates. Asterisks (*) above the bars represent significant differences based on one-way ANOVA with Duncan’s multiple range test (p < 0.05).

As the concentration of BL applied to American ginseng leaves increased, a corresponding decrease in plant growth rate was observed (**Figure 1**). Among all BL-treated groups, plants in the S1 group displayed the highest growth rate. After 21 days of treatment, the S1 group exhibited significant increases of 27.92% in plant height, 17.42% in stem diameter, and 26.70% in leaf area compared with the pre-treatment baseline—all of which were significantly higher than those of the control group (CK) (**Figures 1A, C**). In contrast, the growth rate of plant height in the high-concentration BL treatment groups (e.g., S5 and S6 groups) was significantly lower than that in the CK group (**Figure 1A**). HPLC analysis revealed that neither concentration of BL altered the levels of major ginsenosides Rg1, Rb1, Rb3, Rd, Re, except that the S1 treatment exhibited a certain promoting effect on the synthesis of Rg1 and the S4 treatment had a similar effect on Rd (**Figure 1B**). Therefore, S4 is a typical concentration that can reflect the dual regulatory effects of BL on plant growth and ginsenoside metabolism, rather than a non-specific stress response caused by excessive BL concentrations. Therefore, samples treated with S1 and S4 were used to represent low-concentration and high-concentration BL treatments, respectively, for subsequent omics analysis. These findings indicate that a concentration-dependent regulatory effect of BL on American ginseng growth, without altering the accumulation of primary ginsenosides.

### 3.2 Metabolites in the roots of American ginseng plants under foliar application of brassinolide

**Figure 2:**
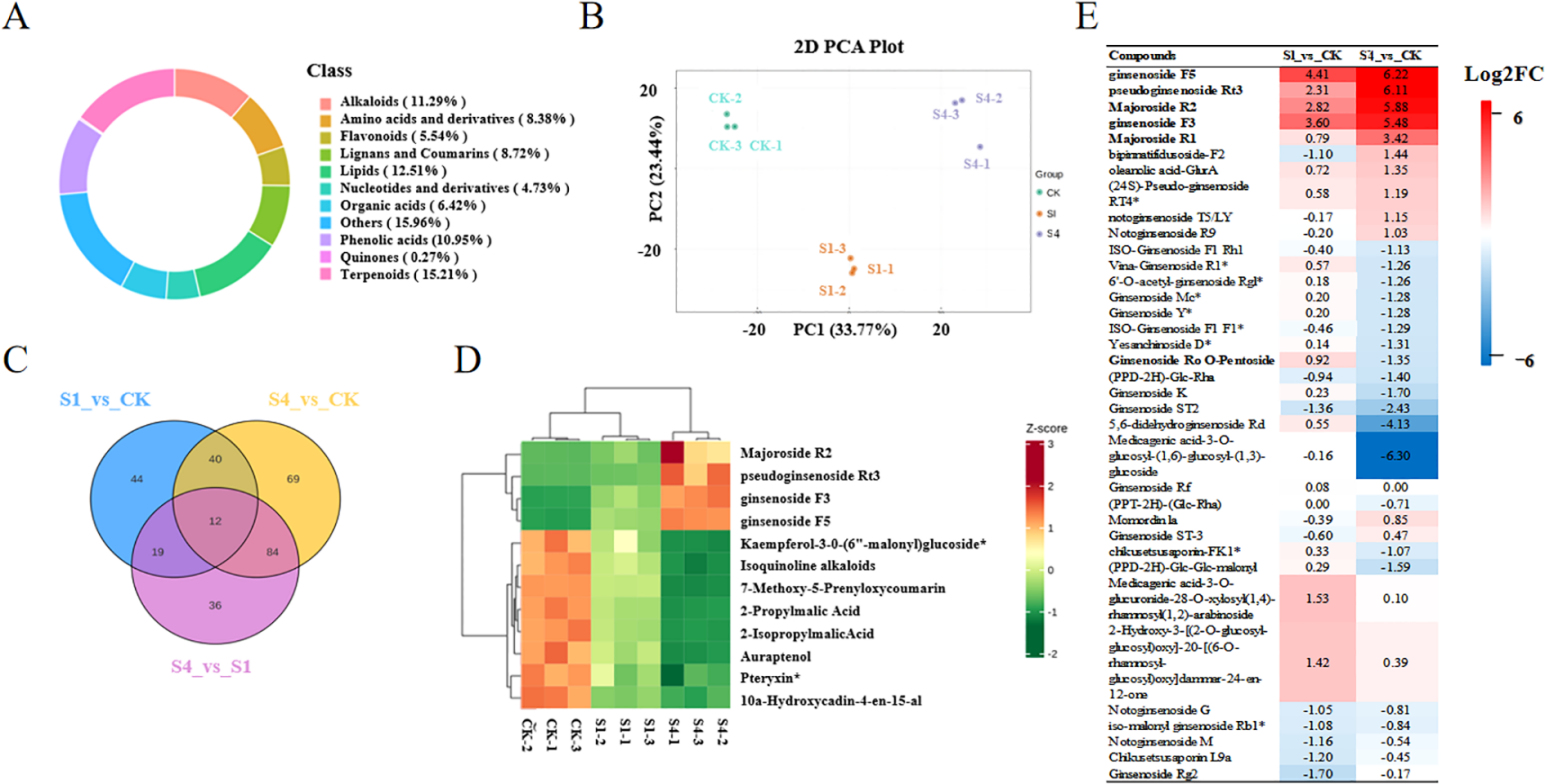
Metabolomic analysis of roots of American ginseng plants under foliar application of brassinolide. **(A)** Circular diagram of metabolite category composition. **(B)** PCA of all metabolite. **(C)** Pairwise compare the DAMs in groups on the Venn diagram. **(D)** Heatmap of the 12 identical DAMs among the three comparison groups. **(E)** Heatmap of the difference ginsenosides among S1_vs_CK and S4_vs_CK.

To investigate the response of American ginseng to different concentrations of BL treatment, UPLC-MS/MS was conducted on root samples subjected to S1 and S4 treatments. A comprehensive analysis identified 1,479 metabolites classified into 11 major groups, with terpenoids representing the largest proportion at 15.21% (**Figure 2A**; **Supplementary Table 1**). Principal component analysis (PCA) revealed distinct metabolic clustering among the S1, S4, and CK groups, with PC1 accounting for 33.77% of the total variance (**Figure 2B**).

A total of 304 differentially accumulated metabolites (DAMs) were successfully identified across all experimental comparisons using stringent screening criteria: variable importance in projection (VIP) ≥ 1 and |log_2_(fold change)| ≥ 1 (**Figure 2C**; **Supplementary Table 2**). Specifically, 205, 115, and 151 DAMs were detected in the S4_vs_CK, S1_vs_CK, and S4_vs_S1 comparisons, respectively (**Figure 2C**). Notably, 12 DAMs were consistently identified across all three comparisons. Among these, four DAMs, ginsenoside F5, pseudoginsenoside Rt3, majoroside R2, ginsenoside F3, were up regulated by BL application, suggesting their potential as robust biomarkers for BL foliar treatment in American ginseng (**Figure 2D**).

Further analysis identified 36 triterpene saponins across the three comparisons (**Figure 2E**; **Supplementary Table 3**). Among them, 5 rare ginsenosides including ginsenoside F5, pseudoginsenoside Rt3, majoroside R2, ginsenoside F3, and majoroside R1 exhibited significant increases of 75-, 69-, 59-, 45-, and 11-fold, respectively, in the S4 group and 21-, 5-, 7-, 12-, and 2-fold in the S1 group (**Figure 2E**; **Supplementary Table 3**). Intriguingly, these rare ginsenosides were ranked among the top 20 DAMs, further highlighting their potential as key biomarkers for the plant’s response to BL treatment (**Supplementary Figure 1**).

**Figure 3:**
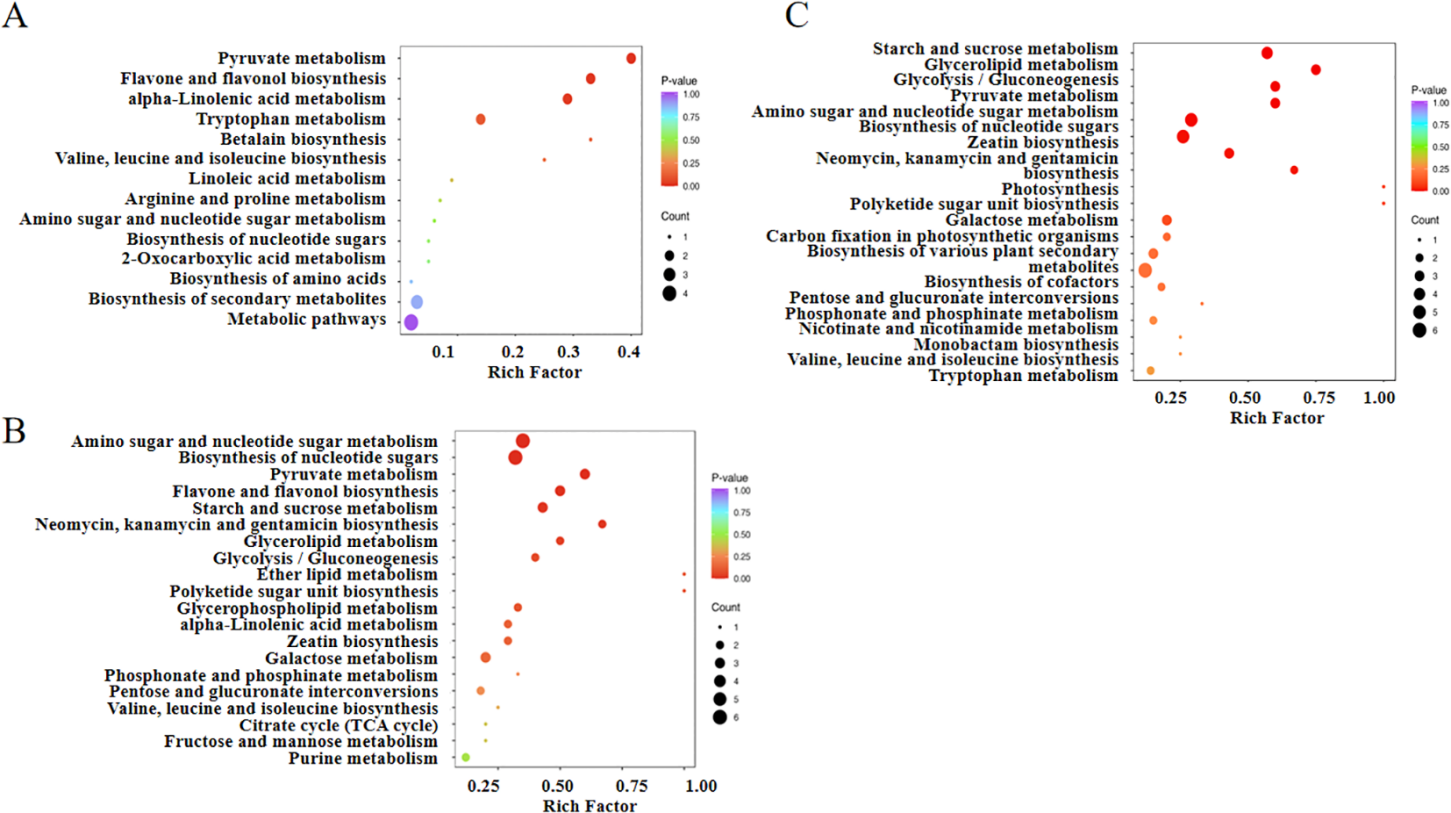
KEGG enrichment analysis of DAMs in the S1_vs_CK **(A)**, S4_vs_CK **(B)** and S4_vs_S1 **(C)** comparison groups.

Meanwhile, the Kyoto Encyclopedia of Genes and Genomes (KEGG) database was used to assign DAMs to metabolic pathways (**Figure 3**). The analysis revealed that the DAMs in S1_vs_CK were primarily enriched in pyruvate metabolism, flavone and flavanol biosynthesis, α-linolenic acid and tryptophan metabolism pathways (**Figure 3A**). DAMs in the S4_vs_CK comparison were mainly associated with amino sugar and nucleotide sugar metabolism, pyruvate metabolism and flavone and flavanol biosynthesis (**Figure 3B**). In the S4_vs_S1, DAMs were primarily involved in starch and sucrose metabolism, glycolipid metabolism, glycolysis/gluconeogenesis, and pyruvate metabolism (**Figure 3C**). These results indicate that BL significantly alters pyruvate and amino sugar/nucleotide sugar metabolism pathways, which are critical for precursor supply in saponin biosynthesis.

### 3.3 Transcriptome analysis of roots of American ginseng plants under foliar application of brassinolide

**Figure 4:**
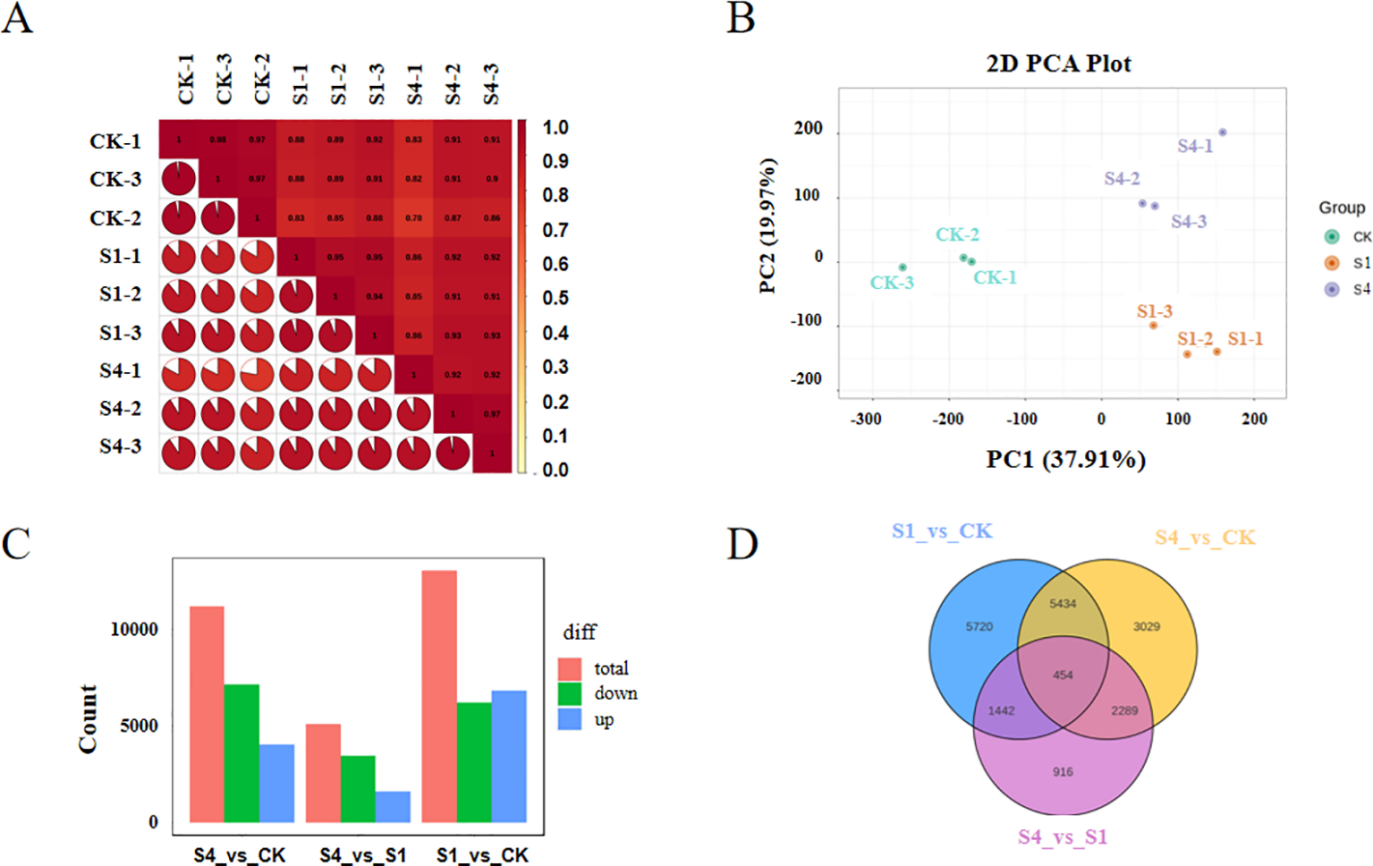
Overview of transcriptome in root of American ginseng plants under foliar applications of BL with different concentration. **(A)** Correlation analysis of transcriptome data of all samples. **(B)** The PCA of transcriptome data. **(C)** Number of DEGs. **(D)** Venn diagram of pairwise comparison of DEGs.

Transcriptome sequencing generated 370698694 clean reads with high quality (Q30 > 89.93%, minimal error rate 0.03%), and over 88% of the reads aligned to the American ginseng genome (**Supplementary Tables 4**, **S5**), validating the suitability of the reference genome employed in this study. A heatmap of sample correlations showed a strong intergroup correlation, with coefficients exceeding 0.78 (**Figure 4A**). PCA separated CK and BL-treated groups along PC1 (37.91% variance; **Figure 4B**). Differential expression analysis identified 19284 DEGs (|log_2_(fold change)| ≥ 1, *p*-value < 0.05) from total mapped 66,874 genes, including 13050 (S1_vs_CK), 11,206 (S4_vs_CK), and 5101 (S4_vs_S1) (**Figure 4C**; **Supplementary Tables 6**, **7**). Venn diagram analysis revealed 454 common DEGs across all comparisons (**Figure 4D**), suggesting the BL-responsive mechanisms is conserved.

Gene Ontology (GO) analyses were conducted to classify the DEGs from the three comparison groups into three major ontologies (biological processes (BP), molecular function (MF), and cellular component (CC)) (**Supplementary Figure 2**). In all pairwise comparisons, the top four BP terms were cellular process, metabolic process, biological regulation, and response to stimulus. Meanwhile, the KEGG database was used to assign DEGs to metabolic pathways (**Supplementary Figure 2**). In the S1_vs_CK comparison, DEGs were significantly enriched in ribosome, ubiquitin mediated proteolysis, ATP-dependent chromatin remodeling and nucleotide excision repair pathways; In the S4_vs_CK comparison, the significantly enrichment was observed in ribosome, ATP-dependent chromatin remodeling, motor proteins, spliceosome pathways. In the S4_vs_S1 comparison, DEGs were significantly enriched in viral life cycle - HIV-1, citrate cycle (tricarboxylic acid cycle, TCA cycle), spliceosome, pyrimidine metabolism pathways. The results indicated that distinct stress adaptation mechanisms were activated under different BL treatment conditions, with S1 treatment primarily influencing protein homeostasis and DNA repair processes while S4 treatment triggered dynamic chromatin reorganization and RNA processing pathways.

**Figure 5:**
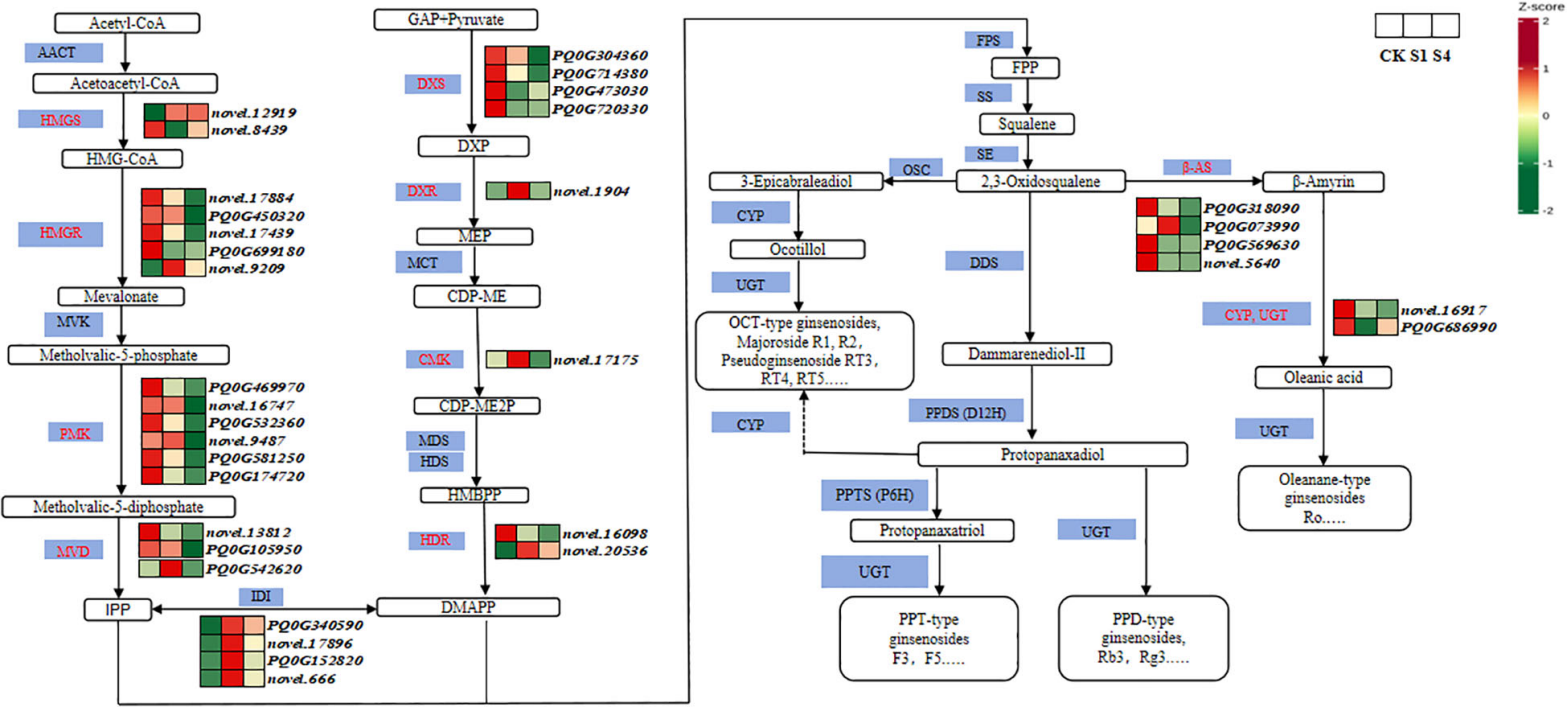
Expression profiles of structural genes involved in the ginsenosides biosynthesis pathway y in roots of American ginseng plants under foliar application of brassinolide. The grids shaded from blue to red represent logarithmic changes in DEGs. Acetoacetyl-CoA thiolase (AACT), Hydroxyethylglutaryl-CoA synthase (HMGS), Hydroxymethylglutaryl-CoA reductase (HMGR), Mevalonate Kinase (MVK), Phosphomevalonate Kinase (PMK), Diphosphomevalonate decarboxylase (MVD), Isopentenyl-diphosphate Delta-isomerase (IDI), 1-deoxy-D-xylulose-5-phosphate synthase (DXS), 1-deoxy-D-xylulose-5-phosphate reductoisomerase (DXR), 2-C-methyl-D-erythritol 4-phosphate cytidylyltransferase (MCT), 4-diphosphocytidyl-2-C-methyl-D-erythritol kinase (CMK), (E)-4-hydroxy-3-methylbut-2-enyl diphosphate synthase (MDS), 1-hydroxy-2-methyl-2-butenyl 4-diphosphate synthase (HDS), 4-hydroxy-3-methylbut-2-en-1-yl diphosphate reductase (HDR), Farnesyl diphosphate synthase (FPS), Squalene Synthase (SS), Squalene Epoxidase (SE), β-amyrin synthase (Beta-AS), Dammarenediol II synthase (DDS), Protopanaxadiol synthase (PPDS), Protopanaxatriol synthase (PPTS), UDP-glycosyltransferase (UGT), Cytochrome P450 oxidase (CYP).

Ginsenoside biosynthesis proceeds through three distinct stages. First, isopentenyl diphosphate (IPP) and its isomer dimethylallyl diphosphate (DMAPP) are produced via the mevalonic acid (MVA) pathway in the cytosol and the methylerythritol phosphate (MEP) pathway in plastids. Subsequently, IPP and DMAPP are sequentially converted into 2,3-oxidosqualene through a series of enzymatic reactions. Finally, diverse ginsenoside structures (e.g., Rh1, Rh2, Rg1, Rg3, Rd, C-K, F2, and Ro) are formed via cyclization, hydroxylation, and glycosylation of 2,3-oxidosqualene. This biosynthetic process is regulated by numerous enzymes encoded by genes such as *AACT*, *HMGS*, *HMGR*, *MVK*, *PMK*, *MVD*, *IDI*, *DXS*, *DXR*, *MEP-CT*, *CDP-MEK*, *MECDPS*, *HMBPPS*, *HMBPPR*, *GPS*, *FPPS*, *SS*, *SOE*, *Beta-AS*, *DDS*, *PPDS*, *PPTS*, *UGT* and *CYP*. The high-level up-regulation of these genes can enhance the flux of ginsenosides biosynthesis pathways. In this study, a total of 34 DEGs were functionally annotated to the ginsenoside biosynthesis pathway (**Figure 5**). Among these, 12 out of 26 DEGs were up-regulated in the S1_vs_CK comparison, while 4 out of 25 DEGs were up-regulated in the S4_vs_CK comparison, indicating that BL treatment affects the gene expression of ginsenosides biosynthesis.

### 3.4 Integrated analysis of the transcriptome and metabolome of the DEGs and DAMs involved in ginsenosides biosynthesis pathways

A combined transcriptomic and metabolomic analysis was conducted to further elucidate the mechanism underlying the response of American ginseng to BL treatment. The KEGG analysis revealed that the differentially expressed genes (DEGs) and differentially accumulated metabolites (DAMs) identified in the S4_vs_CK co-enriched in neomycin, kanamycin and gentamicin biosynthesis pathway (ko00524) (**Supplementary Figure 3A**). In the S4_vs_S1 comparison, DEGs and DAMs were co-enriched in pyruvate metabolism (ko00620) and amino sugar/nucleotide sugar metabolism (ko00520) (**Supplementary Figure 3B**). However, no significant co-enrichment of DEGs and DAMs were observed in any pathway in the S1_vs_CK comparison (**Supplementary Figure 3C**).

Notably, in both the S4_vs_CK and S4_vs_S1 comparisons, the genes *PQ0G073990*, *PQ0G318090*, *novel.16917*, *PQ0G686990*, along with the metabolites phosphoenol pyruvate and ginsenoside Ro O-Pentoside, were concurrently enriched in the oleanane-type saponin biosynthesis pathway (**Figure 5**). The *β* - amyrin synthase encoding genes *PQ0G073990* and *PQ0G318090*, and *β* - amyrin 28 - monooxygenase encoding gene *Novel.16917*, were down-regulated in the S4 treatment group. The down-regulation pattern was consistent with the reduced levels of ginsenoside Ro O-Pentoside. Therefore, *PQ0G073990*, *PQ0G318090*, *Novel.16917* and *PQ0G686990* are considered key candidate genes responsive to BL stimulation and involved in the regulation of the ginsenoside Ro biosynthesis.

### 3.5 Weighted gene co-expression network analysis

**Figure 6:**
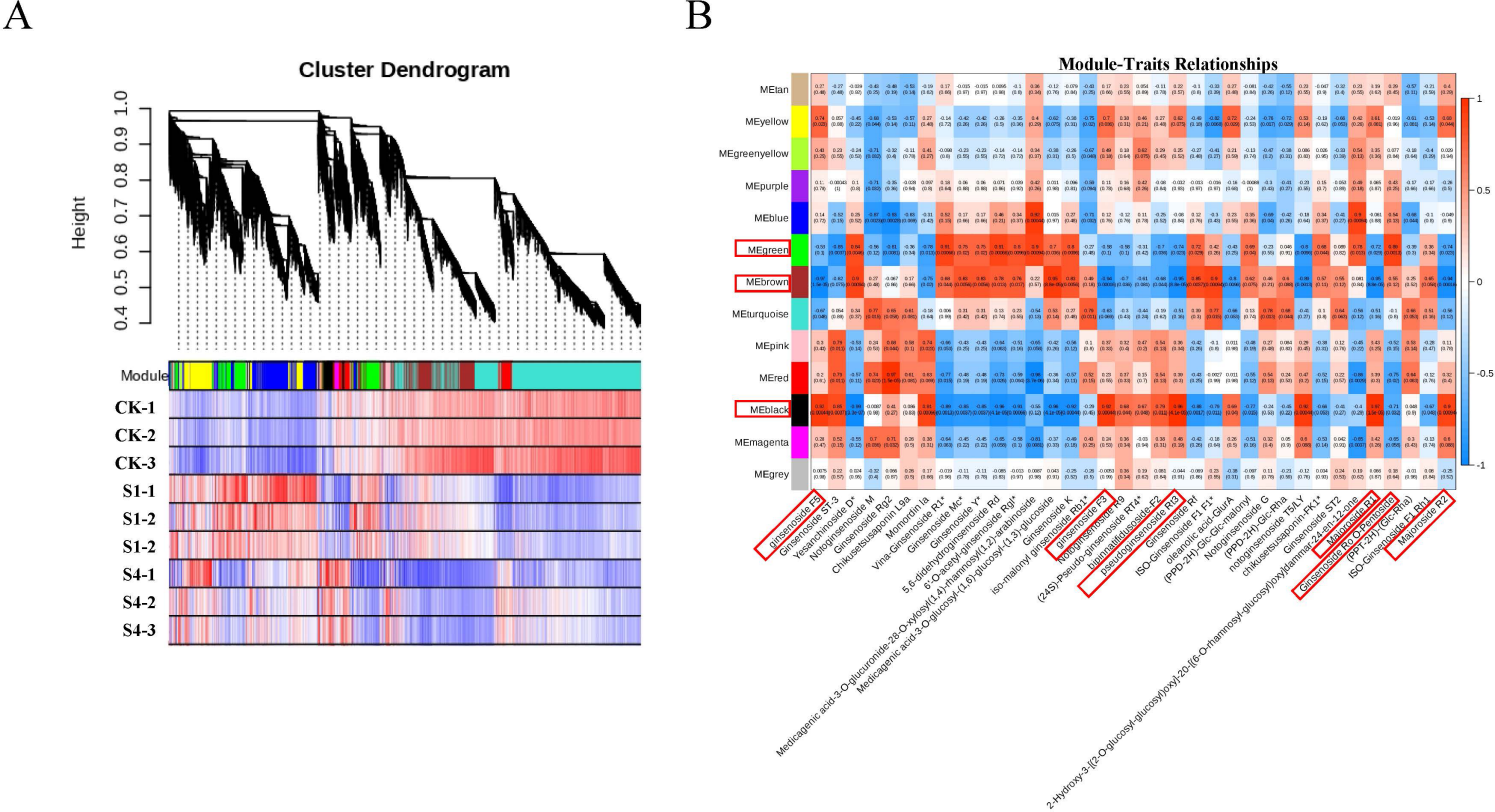
Weighted gene co-expression network analysis (WGCNA). **(A)** Dendrogram of the co-expression modules (clusters). The major tree branches constitute 13 modules labeled with different colors. **(B)** Heat map showing the correlations between modules and ginsenosides compounds. Red indicates a positive correlation, and blue indicates a negative correlation between the cluster and the sample.

Weighted gene co-expression network analysis (WGCNA) identified 13 distinct gene modules (**Figure 6A**). Among them, genes in the black module were significant up-regulated in the S4 group and exhibited strong positive correlations (|*r*| > 0.85) with 5 rare ginsenosides, i.e., ginsenoside F5, ginsenoside F3, pseudoginsenoside Rt3, majoroside R1 and majoroside R2. In contrast, genes in the brown module were significantly down-regulated in the S4 group and showed strong negative correlations (|*r*| > 0.85) with these same ginsenosides (**Figure 6B**). Genes in the green module were significantly down-regulated in the S1 group and up-regulated in S4 group and demonstrated strong positive correlations (|*r*| > 0.85) with ginsenoside Ro O-Pentoside, which increased in the S1 group and decreased in the S4 group (**Figure 6B**).

**Figure 7:**
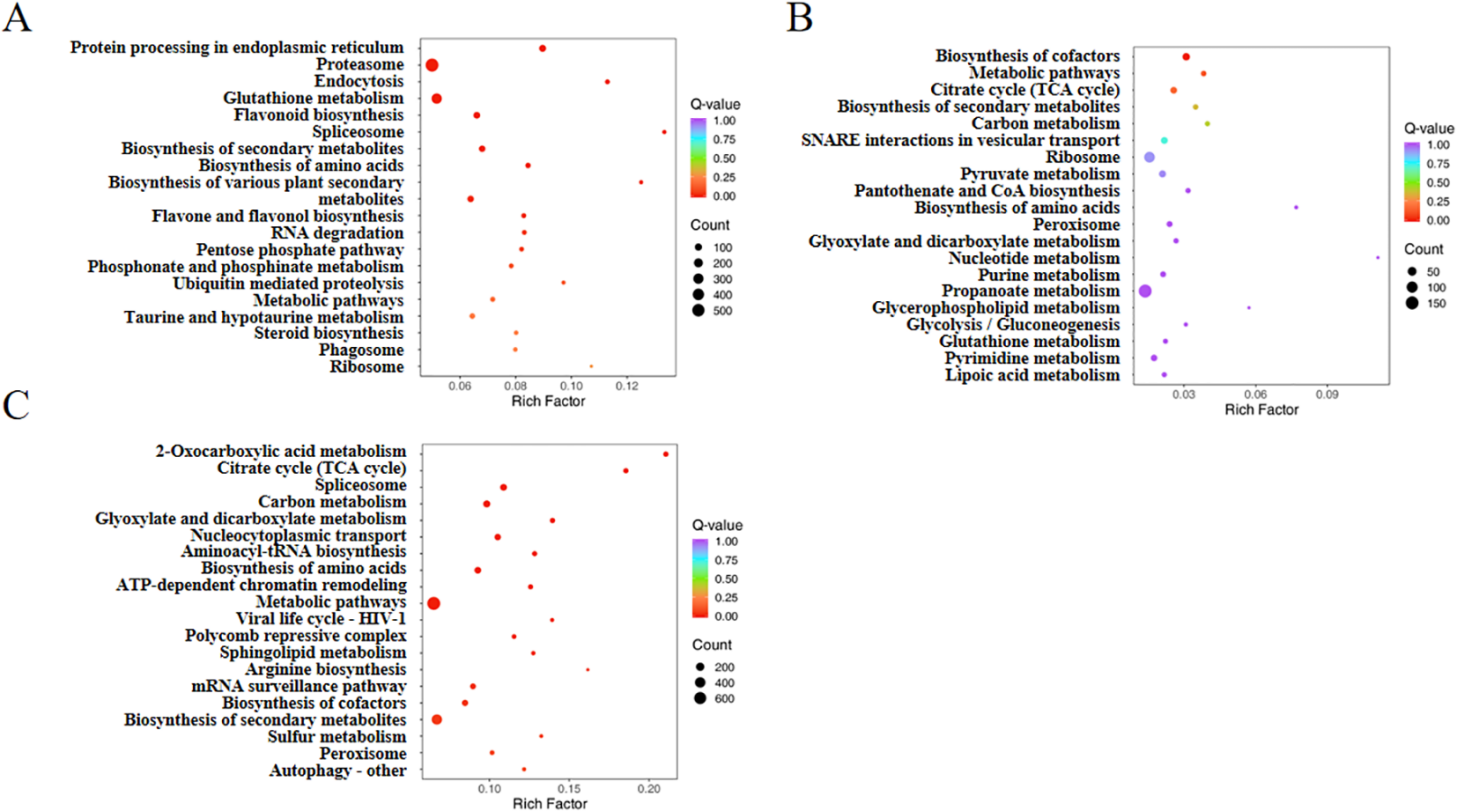
KEGG pathway enrichment of genes in three modules. **(A)** KEGG enrichment in black model; **(B)** KEGG enrichment in brown model, and **(C)** KEGG enrichment in green model. Presented are the top 20 most significantly enriched pathways.

The KEGG analysis showed that the black module genes were enriched in ‘Protein processing in endoplasmic reticulum’, ‘proteasome’, ‘endocytosis’, ‘glutathione metabolism’ (**Figure 7A**); the brown module genes were enriched in 2-oxocarboxylic acid metabolism, citrate cycle (TCA cycle), spliceosome, carbon metabolism biological processes (**Figure 7B**); the green module genes were enriched in biosynthesis of cofactors, metabolic pathways, citrate cycle (TCA cycle), biosynthesis of secondary metabolites biological processes (**Figure 7C**). The result indicated that BL can promote the accumulation of five rare ginsenosides and the degradation of ginsenoside Ro O-Pentoside by regulating multiple key metabolic pathways and regulatory networks.

### 3.6 BR-specific regulatory networks analysis

To investigate the effect of exogenous BR on the BR signaling pathway in *Panax quinquefolius* roots, we identified DEGs enriched in the BR signaling pathway from the transcriptome data. A total of 118 DEGs were mapped to this pathway, including BRI1 (receptor), BAK1 (co-receptor), BSK (BR-signaling kinase, a signal transducer), BKI1 (BRI1 kinase inhibitor 1, a negative regulator), BIN2 (BR-insensitive 2, a negative regulator), BZR1/BES1 (transcription factors), and downstream response genes such as TCH4 (cellulose synthase-like gene) and CYCD3 (cyclin D3 gene) (**Supplementary Figure 4**). These DEGs covered the entire BR signaling cascade, spanning three critical stages: receptor recognition, intracellular signal transduction, and downstream target gene response(**Supplementary Figure 4**). Notably, comparative analysis between BR-treated groups (S1 and S4) and the control group (CK) revealed that the expression of most of these 118 DEGs was down-regulated (**Supplementary Figure 4**). This finding suggests that exogenous BR application to the aerial parts of *P. quinquefolius* modulates the BR signal transduction pathway in the roots.

Plant hormones can regulate ginsenoside biosynthesis by modulating the activity of key enzymes and the expression of their encoding genes in the ginsenoside biosynthetic pathway. Among these enzymes, cytochrome P450 monooxygenases (CYP450) and UDP-glucosyltransferases (UGT) play indispensable roles in the late-stage modification of ginsenoside aglycones (Han et al., 2011, 2012; Li et al., 2024a). In this study, we identified 91 CYP450 and 30 UGT as DEGs using the transcriptome data (**Supplementary Table 8**). The KEGG pathway enrichment analysis showed that these DEGs were significantly enriched in seven metabolic pathway: biosynthesis of secondary metabolites (ko01110), brassinosteroid biosynthesis (ko00905), biosynthesis of various plant secondary metabolites (ko00999), zeatin biosynthesis (ko00908), cutin, suberine and wax biosynthesis (ko00073), and sesquiterpenoid and triterpenoid biosynthesis (ko00909) (**Supplementary Figure 5A**). The result indicate that exogenous BR treatment on *P. quinquefolius* leaves alters the expression of genes encoding CYP450 and UGT in the roots, which may directly or indirectly regulate the biosynthesis of rare ginsenosides, brassinosteroid, zeatin, cutin, suberine and wax.

### 3.7 Potential candidate genes in the downstream of ginsenoside biosynthesis pathway

**Figure 8:**
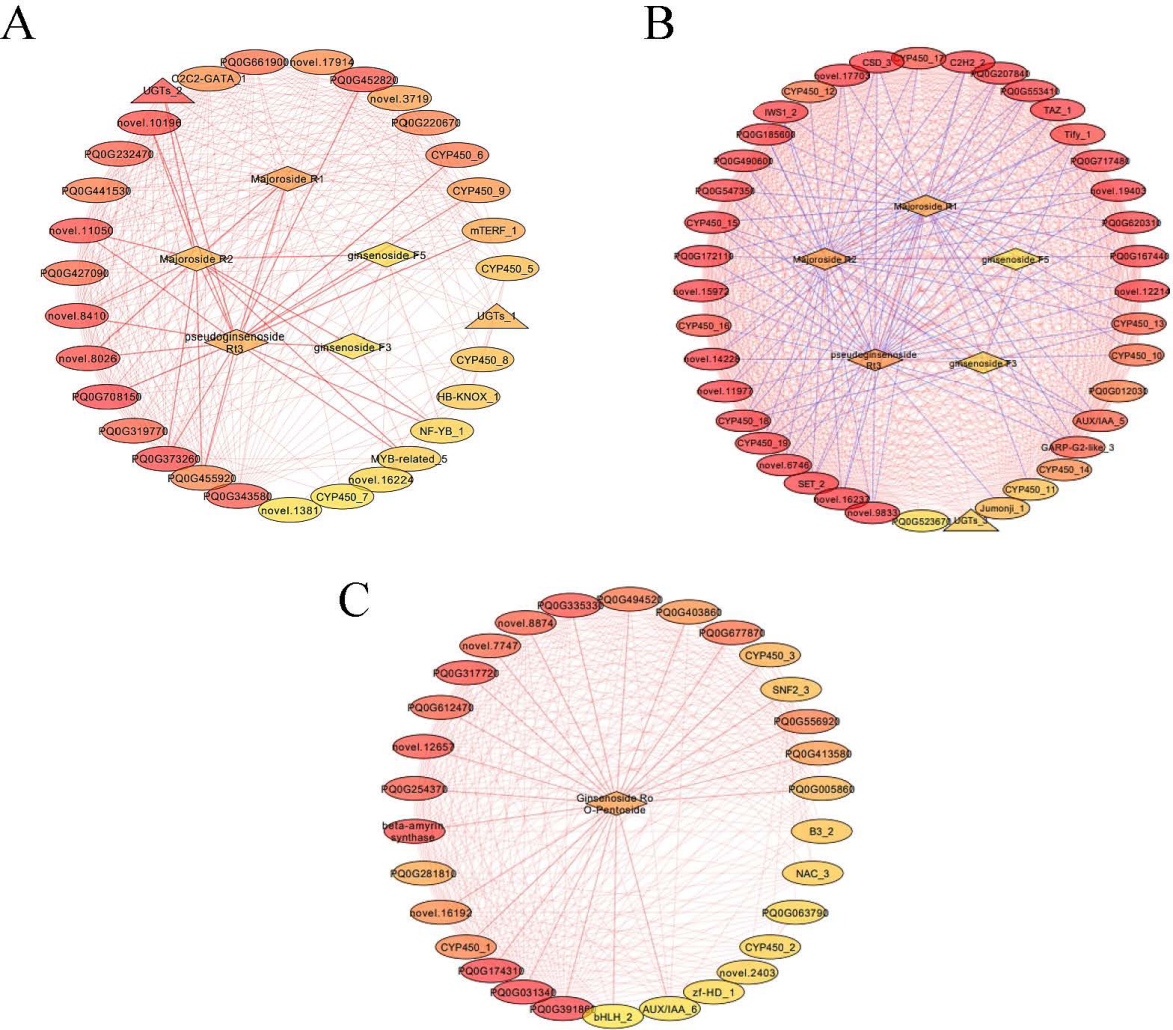
Correlation network of the gensinosides and candidate genes in black **(A)**, brown **(B)**, and green **(C)** modules visualized by the Metware Cloud. In **(A–C)** orange and blue edge color indicate positive and negative correlation, respectively. and the degree of connectivity. The node size represents the degree of connectivity.

We identified 3 gene expression modules associated with rare ginsenoside biosynthesis via WGCNA. Furthermore, we identified 19 CYP450 genes and 3 UGT genes from these three modules as candidate genes involved in the regulation of rare ginsenosides (**Supplementary Figure 5B**). Additionally, we identified 184 transcription factors (TFs) from these models and prioritized the top 10 TFs with the highest connectivity (kwithin values) as putative regulators of pathway genes (**Figure 8**). The KEGG analysis of these 206 genes showed that, these genes were significantly enriched in three key pathways: plant hormone signal transduction (ko04075), brassinosteroid biosynthesis (ko00905), and biosynthesis of various plant secondary metabolites (ko00999). Among them, the plant hormone signal transduction pathway contained 22 DEGs, which were involved in six major plant hormone signaling pathways. These results demonstrate that exogenous BR treatment on *P. quinquefolius* leaves alters the expression of genes involved in hormone biosynthesis and hormone signal transduction pathways in the roots. Given the crosstalk between plant hormones and secondary metabolism, this transcriptional reprogramming may indirectly regulate the biosynthesis of rare ginsenosides by modulating hormone homeostasis and signaling cascades.

To elucidate gene-metabolite associations, pearson correlation analyses were performed using the aforementioned candidate genes, the top 10 TFs, the top 20 genes with the highest intramodular connectivity (kwithin value) across the three modules, and 6 rare ginsenosides (ginsenoside F5, pseudoginsenoside Rt3, majoroside R2, ginsenoside F3, majoroside R1 and ginsenoside Ro O-Pentoside). A co-expression network was constructed based on significant correlations (|cor| > 0.85, *p* < 0.05). Visualization of this network using the Metware Cloud Platform enabled the identification of key regulatory nodes involved in ginsenoside biosynthesis. The co-expression network analysis revealed that 16 CYP450/UGT i.e., *UGTs_2*, *CYP450_6*, *CYP450_9*, *CYP450_12*, *CYP450_15*, *CYP450_16*, *CYP450_18*, *CYP450_19*, *CYP450_11*, *CYP450_14*, *CYP450_10*, *CYP450_13*, *CYP450_17*, *CYP450_1*, *CYP450_3*, and *CYP450_4*, and 17 TFs such as NF-YB, MYB_related, mTERF, bHLH, CSD, IWS1, SET, Jumonji, GARP-G2-like, Tify, TAZ, C2H2, SNF2 and AUX/IAA were strongly correlated (|*r*| > 0.85) with 6 rare ginsenosides (**Supplementary Table 9**). Six genes were randomly selected from above candidate genes and used to verify the accuracy of the transcriptomes by qRT-PCR, which showed consistency with the RNA-seq findings (**Supplementary Figure 6**). These genes are likely to play pivotal roles in BL-induced biosynthesis or degradation of 6 rare ginsenosides in American ginseng and warrant further investigation.

## 4 Discussion

Brassinosteroids (BRs), are well-documented phytohormones that regulate plant growth, stress adaptation, and secondary metabolism across diverse species (Mao et al., 2017; Guo et al., 2022; Han et al., 2023; Zhang et al., 2023a). However, their role in modulating ginsenoside biosynthesis in *Panax quinquefolius* (American ginseng) remains underexplored. Metabolite profiling and transcriptomic analysis are convenient and effective methods to investigate the biosynthesis of key metabolites in different developmental stages of plants or under different treatment conditions (Bai et al., 2021; Zhang et al., 2022; Ran et al., 2023; Zhang et al., 2025). In this study, we integrated metabolomic and transcriptomic analyses to uncover the concentration-dependent effects of foliar brassinolide (BL) application on plant growth and ginsenoside accumulation in the roots of American ginseng. Our findings offer novel insights into the regulatory mechanisms underlying BL-mediated modulation of secondary metabolism in this important medical plant.

### 4.1 BL modulates growth-ginsenoside synergy in a dose-dependent manner

Foliar application of BL has been widely reported to enhance plant vegetative growth, increases yield, and improves the quality of agricultural products (Ali et al., 2022; Zhang et al., 2023a). In the present study, treatment with low-concentration BL significantly promoted plant height, stem diameter, and leaf area. In contrast, high-concentration BL showed no growth-promoting effects. These findings support a concentration-dependent, threshold effect in BL-mediated vegetative growth regulation. This phenomenon is consistent with the dual regulatory mechanism of the BR signaling pathway. At optimal concentrations, BR is known to activate auxin synthesis and transport, thereby enhancing cell elongation and division. However, at higher concentrations, feedback inhibition or increased metabolic burden may suppress, resulting in diminished physiological benefits (Mao et al., 2017).

Previous studies have indicated that BL significantly increase the accumulation of notoginsenoside R1 content in *Panax notoginseng*, but had no significant effect on Rg1, Rb1, Rd and Re (Yu et al., 2020). Similarly, Zhou et al. (2003) reported that the lower concentrations of BL stimulated saponin accumulation, whereas the higher concentrations of BL inhibited this process. Zheng et al. (2025) found that BR-induced BZR1 regulate vegetative development and flavonoids biosynthesis in *Scutellaria baicalensis*. Consistent with these observations, our study demonstrated that neither low nor high concentrations of BL altered the accumulation of major ginsenosides (Rb1, Rb2, Rb3, Rd, Re) (**Figure 2**), indicating the weak effect of BL on the major ginsenosides in American ginseng.

### 4.2 Metabolomic insights into BL-induced rare ginsenoside synthesis and metabolic flux redistribution

Non-targeted metabolomics identified a total of 1479 metabolites, with terpenoids (15.21%), lipids (12.51%), and alkaloids (11.29%) as the main categories. Principal component analysis (PCA) showed distinct clustering among the control group and BL-treated groups, indicating that BL application significantly alters the global metabolic landscape. A total of 304 differentially accumulated metabolites (DAMs) were identified using VIP ≥ 1 and |log_2_(fold change)| ≥ 1 as criteria, including 36 triterpenoid saponins, especially 5 rare ginsenosides i.e., ginsenoside F5, pseudoginsenoside Rt3, majoroside R2, ginsenoside F3, majoroside R1-were dramaticallyupregulated by 11 to 75 fold.

These rare ginsenosides have been extensively studied for their remarkable antitumor and immunomodulatory properties (Nguyen et al., 2010; Tran et al., 2014; Li et al., 2016). Previous studies have demonstrated that compounds such as ginsenoside F5, ginsenoside F3, and pseudoginsenoside Rt3, exhibited potent antitumor and immunomodulatory activities. These ginsenosides have been shown to inhibit the proliferation of human leukemia HL - 60 cells through the apoptosis induction,and to enhance immune responses in murine spleen cells by modulating the cytokine production and gene expression (Yu et al., 2004). Such findings not only highlight the potential of these rare ginsenosides as bioactive agents but also underscore the efficacy of the BRs in promoting the biosynthesis of these valuable metabolites.

Notably, low-concentration BL promotes the vegetative growth and induce accumulation of the 5 rare ginsenosides (2–21-fold). In contrast, high-concentration BL did not enhance plant growth but resulted in a more accumulation of these rare ginsenosides (11–75-fold increase). These findings suggest a concentration-dependent reprogramming of metabolic flux: at lower BL levels, carbon and energy resources are preferentially allocated toward primary metabolism to support vegetative growth, whereas higher BL levels divert metabolic flux toward secondary metabolic pathways-particularly for rare ginsenoside biosynthesis. This hypothesis is further supported by KEGG pathway enrichment analysis, which indicated that increasing BL concentration alters central carbon metabolism. At low concentrations, carbon sources pathways associated with primary growth, while at high concentrations, these resources are redirected toward secondary metabolism (rare ginsenosides). This redistribution of metabolic flux offers a molecular explanation for the concentration-specific effects of BL on growth and metabolite accumulation.

The phenomenon observed aligns with the well-established growth-metabolism synergy in medicinal plants, where resource allocation is balanced between the biomass accumulation and secondary metabolite production. For example, BR treatment synergistically enhances both tuber yield and total alkaloid content in *Pinellia ternata* (Guo et al., 2022). These insights provide a promising strategy for the targeted enhancement of pharmacological valuable rare ginsenosides through optimized BL application. It support a alternative method in improving the quality and therapeutic potential of American ginseng for use in functional foods, health supplements and pharmaceutical formulations.

### 4.3 Transcriptomic reprogramming driving BL-mediated metabolic shifts

Transcriptome sequencing yielded 370698694 high-quality reads (Q30 > 89.93%), with over 88% of the reads successfully mapped to the American ginseng reference genome, indicating a high level of sequencing depth and data reliability. Principal component analysis (PCA) showed clear separation between the control (CK) and BL-treated samples, demonstrating that foliar application of BL significantly alters the transcriptomic landscape of American ginseng roots. A total of 19284 DEGs were identified across all BL treatment comparisions, indicate a broad and dynamic transcriptional response to BL application. 454 were shared across all treatment groups, suggesting the existence of conserved core regulatory genes involved in BL signal perception and transduction. These common DEGs likely represent central components of the BL response network, potentially acting as key transcriptional hubs or signaling regulators.

KEGG pathway enrichment of DEGs between high- and low-concentration BL treatments (S4_vs_S1) revealed significant enrichment in pathways such as the viral life cycle-HIV-1 (which may be related to stress-responsive gene expression patterns), citrate cycle (TCA cycle), spliceosome, and pyrimidine metabolism. The TCA cycle is a pivotal metabolic pathway that governs ATP production and provides intermediates for various biosynthetic processes. The altered expression of genes within this pathway indicates that high BL concentrations may reprogram the plant’s energy metabolism, potentially redirecting carbon fluxes towards secondary metabolism, as supported by the enhanced accumulation of rare ginsenosides observed in the metabolomic analysis.

Interestingly, although metabolomic data showed that the accumulation of rare ginsenosides was significantly increased under high-concentration BL treatment, most genes in the ginsenoside biosynthetic pathway (including most mevalonate (MVA) pathway genes) were significantly down-regulated at the transcriptional level. This apparent contradiction can be jointly explained by metabolic reprogramming in plants under phytotoxic stress and the complexity of multilevel regulation in ginsenoside biosynthesis. Plant hormones are essentially regulatory molecules. However, improper application—such as excessively high concentrations or unsuitable timing—can convert them into exogenous chemical stressors, inducing phytotoxic stress responses in plants. Under such conditions, plants activate a resource reallocation strategy and suppress growth-related primary metabolism (represented by the down-regulation of most MVA pathway genes) to reduce energy consumption. Meanwhile, post-transcriptional and post-translational regulatory mechanisms (e.g., mRNA stability control, enzyme phosphorylation or glycosylation) can enhance the activity of key biosynthetic enzymes even at reduced transcriptional levels, thus sustaining the synthesis of defensive secondary metabolites. Furthermore, BL may induce the expression of specific cytochrome P450 (CYP450) monooxygenases and UDP-glycosyltransferases (UGTs). These enzymes are known to mediate the structural diversification and glycosylation of rare ginsenosides, and can redirect metabolic flux toward specific branches of the biosynthetic pathway, thereby promoting the production of rare ginsenosides.

### 4.4 Multi-omics integration reveals key genes and regulatory networks of BL action

The integrated transcriptomic and metabolomic analysis showed that DEGs and DAMs in the S4_vs_CK comparsion were co-enriched in neomycin, kanamycin, and gentamicin biosynthesis pathways (ko00524), while those in the S4_vs_S1 group were enriched in pyruvate metabolism and amino sugar metabolism. These findings further support the notion that high-concentration BL treatment induces a substantial reprogramming of carbon metabolism, redirecting metabolic fluxes to favor the biosynthesis of rare ginsenoside. Previous studies have shown that CYP716A subfamily members serve as multifunctional oxidases in triterpenoid biosynthesis (Carelli et al., 2011; Fukushima et al., 2011; Han et al., 2013; Tamura et al., 2017). Tian et al. (2022) reported that the *PscCYP716A1* was transcriptionally up-regulated by Cd stress but suppressed by the exogenous brassinolide (BR). In our study, high-concentration BL treatment resulted in the down-regulation of *β*-amyrin synthase genes (*PQ0G073990*, *PQ0G318090*, *PQ0G569630*, novel.5640), and CYP450 (*novel.16917* and *PQ0G686990*), which corresponded with a decrease in oleanane-type saponin ginsenoside Ro. This suggests that BL may redirect the metabolic flux from the biosynthesis of oleanane-type toward PPD/PPT/OCT-type rare ginsenosides.

Long-distance inter-organ communication is fundamental for plants to adapt to fluctuating environments. As core signaling molecules, most plant hormones (abscisic acid, auxin, cytokinin, and jasmonic acid) are translocated long-distance via the vascular system in their intact forms, while gibberellin and ethylene rely on precursor translocation (Zhang et al., 2023b). However, BRs have long been considered to act only locally or in an autocrine manner due to their low mobility, and no long-distance BR transport has been detected in pea grafting and ³H-BR feeding experiments (Symons and Reid, 2004), which is consistent with the general consensus that BRs do not undergo long-distance translocation. Notably, hormones do not function independently; extensive signaling crosstalk occurs between BRs and other mobile systemic signals—including reactive oxygen species (ROS), endogenous hormones, sugars, small RNAs, mRNAs, peptides, second messengers, and proteins—which can be transported via the vascular system to distal organs (e.g., roots) to exert systemic regulatory effects (Notaguchi and Okamoto, 2015; Ye et al., 2025) (**Figure 3**). These signals are proposed to mediate the leaf-to-root communication after foliar BL application: for instance, leaf-derived ROS waves can be transmitted to roots via the Ca²^+^-RBOH-ROS module (Li et al., 2025); sucrose, as a mobile signal and energy substrate, can modulate distal organ responses via crosstalk with gibberellin metabolism (Su et al., 2024); and miR395/miR399, translocated from shoots to roots, can regulate root physiological processes (Zhao and Zheng, 2025). These systemic signals may act synergistically to transmit the BL-induced leaf signal to the roots.

Previous studies have identified diverse transcription factors (TFs) regulating ginsenoside biosynthesis in *Panax* species, primarily belonging to the bHLH, AP2/ERF, bZIP, WRKY, and NAC families (Mohanan et al., 2023). These include *PnbHLH1*, *PqWRKY1*, *PnWRKY1*, *PgWRKY4X*, *PnERF1*, *PgMYB2*, *PnPHL8*, and *PgNAC72*, which modulate the expression of key biosynthetic genes such as *FPS*, *SS*, *SE*, *DS*, and *CYP716A47* (Zheng et al., 2023). Among these regulators, *PqWRKY1*, *PnERF1*, and *PgMYB2* are directly induced by jasmonic acid (JA) signaling, linking hormone perception to the activation of ginsenoside biosynthetic pathways. In this study, 17 differentially expressed transcription factors (TFs) were identified, belonging to 15 families, whose functional regulation is closely associated with hormone signaling pathways and triterpenoid saponin metabolism. Among them, *Tify_1*, *bHLH_2*, and *MYB-related_5* are all involved in jasmonic acid (JA) signal transduction. Collectively, these results indicate that Tify_1, bHLH_2, and MYB-related_5 may serve as key regulators of ginsenoside biosynthesis, providing novel targets for further functional validation and genetic improvement of ginsenoside production in *Panax* plants.

These key regulatory nodes offer new insights into the molecular mechanisms of ginsenoside biosynthesis and suggest a complex regulatory network by which BL modulates secondary metabolism. These genes may play crucial roles in modulating the biosynthesis of rare ginsenosides in response to varying BL concentrations.

### 4.5 Research implications and application prospects

This study systematically elucidated the dual regulatory mechanism of foliar-applied BL on American ginseng, whereby low concentrations promote growth, while both low and high concentrations activated rare ginsenoside synthesis via transcriptional reprogramming and transcription factor (TF) networks. The study-identified 16 CYP450/UGT genes, along with 17 TFs, provide direct targets for metabolic engineering. These targets can help can overcome traditional cultivation bottlenecks through microbial heterologous expression. Additionally, BL’s concentration-dependent precise regulation strategy offers a new direction for quality improvement of American ginseng—low doses optimize biomass, and high doses directionally enhance medicinal components. Future research should further dissect the interaction mechanisms between core TFs and CYP450/UGT genes, and thereby provide theoretical support for the synthetic biology production of rare ginsenosides and sustainable agriculture.

### 4.6 Research limitations and future perspectives

In this study, phenotypic measurements and metabolomic analysis revealed that brassinolide (BL) promotes the growth of *Panax quinquefolius* L. and increases the content of rare ginsenosides in the roots. Furthermore, conjoint analysis of metabolomics and transcriptomics was performed to screen candidate genes involved in BL-regulated biosynthesis of rare ginsenosides in *Panax quinquefolius*.

However, this study has several limitations: the candidate genes (16 CYP450/UGT genes and 17 transcription factors) and key regulatory nodes identified by multi-omics association analysis have not been functionally validated; the signal transduction mechanism underlying the regulation of root secondary metabolism by foliar application of BL remains incompletely elucidated; the fold changes of rare ginsenosides were obtained from untargeted metabolomic relative quantification, which needs to be further verified by absolute quantification using targeted LC-MS/MS with authentic standards.

Future research can focus on the following aspects: functionally verifying the candidate genes via techniques such as gene overexpression and knockout to clarify their specific roles in the biosynthesis of rare ginsenosides; performing absolute quantification of rare ginsenosides using targeted LC-MS/MS to confirm their content variations; further identifying the long-distance signaling factors of brassinosteroids (BR) in *Panax quinquefolius* roots to reveal the in-depth molecular mechanism by which BL regulates rare ginsenoside biosynthesis.

## Data Availability

The original contributions presented in the study are publicly available. This data can be found here: NCBI, PRJNA1481795.
