# Supplementary material for: Metabolomics combined with transcriptomics analysis on ginsenosides accumulation in root of American ginseng plants under foliar applications of brassinolide
Source: Front Plant Sci. 2026 Jul 3;17:1798852. doi: 10.3389/fpls.2026.1798852 (PMC13378128; doi:10.3389/fpls.2026.1798852)
Supplement: Supplementary file 2 [file Image1.pdf]

frontiers

Supplementary Material

Supplementary Figures

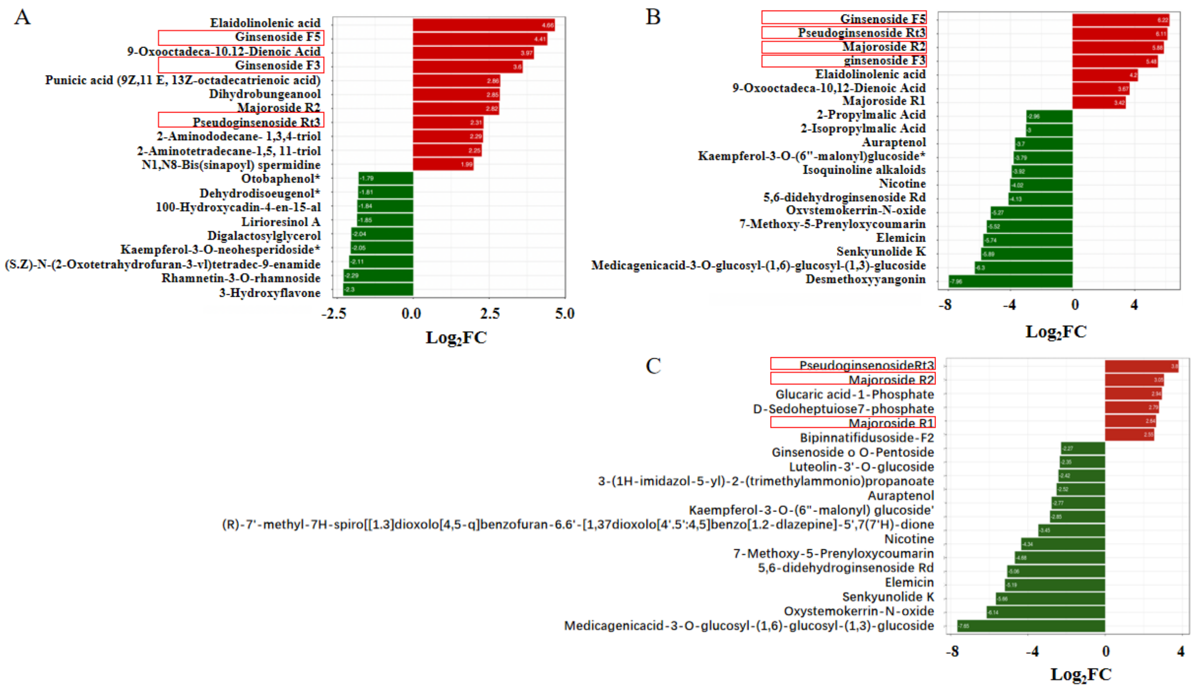

Figure S1 The top 20 DAMs identified in S4_vs_CK **(A)**, S4_vs_S1 **(B)** and S1_vs_CK **(C)**.

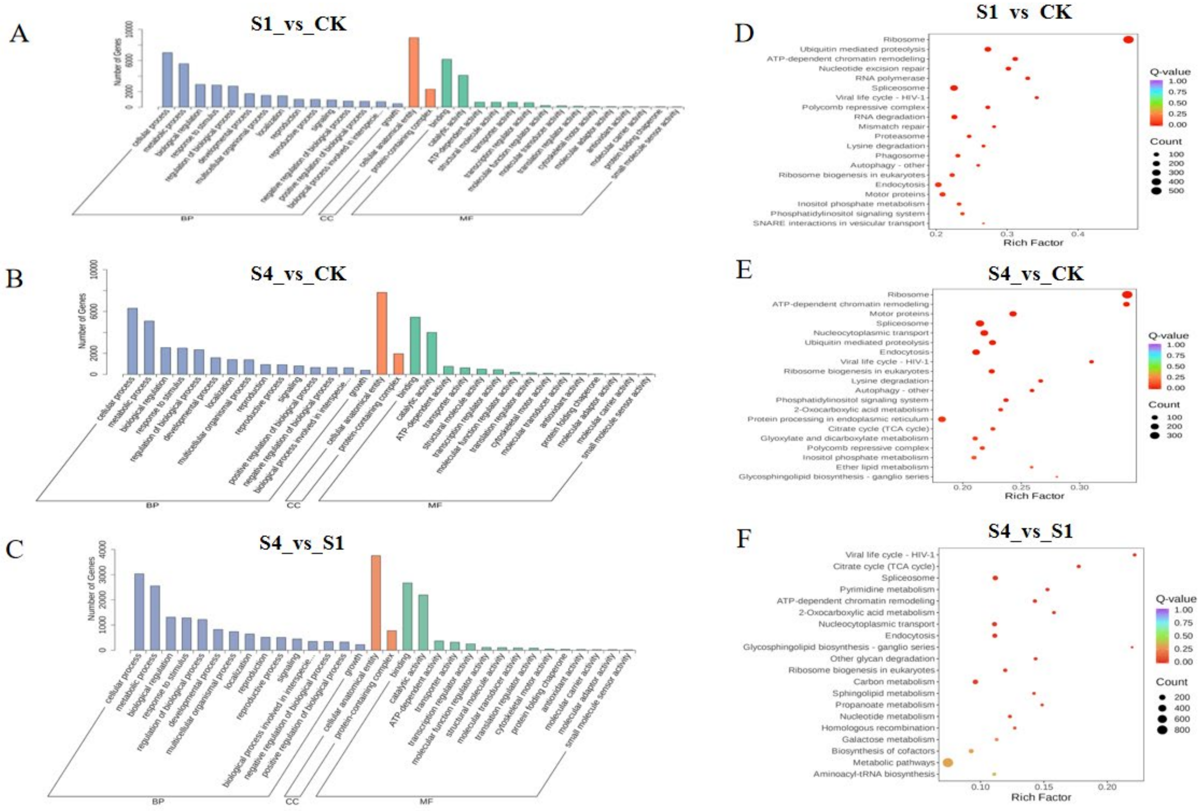

Figure S2 Functional categories shared by three comparison groups identified through Gene Ontology (GO) enrichment analysis **(A - C)** and Kyoto Encyclopedia of Genes and Genomes KEGG **(D - F)** enrichment analysis using transcriptome data.

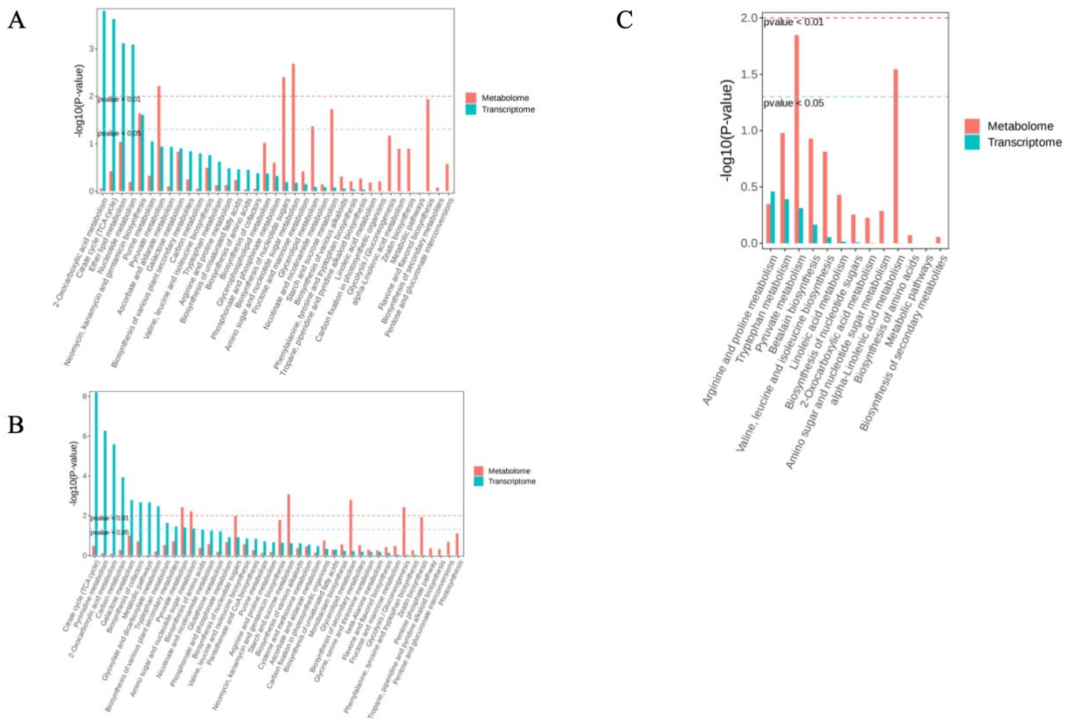

Figure S3 KEGG pathway enrichment analysis of DEGs and DAMs identified in S4_vs_CK **(A)**, S4_vs_S1 **(B)** and S1_vs_CK **(C)**. The abscissa represents metabolic pathways, and the ordinate represents the enriched *p*-value of DEGs (white) and DAMs (blue), which is represented by －log (*p*-value) using a threshold of $p < 0.01$ and $p < 0.05$.

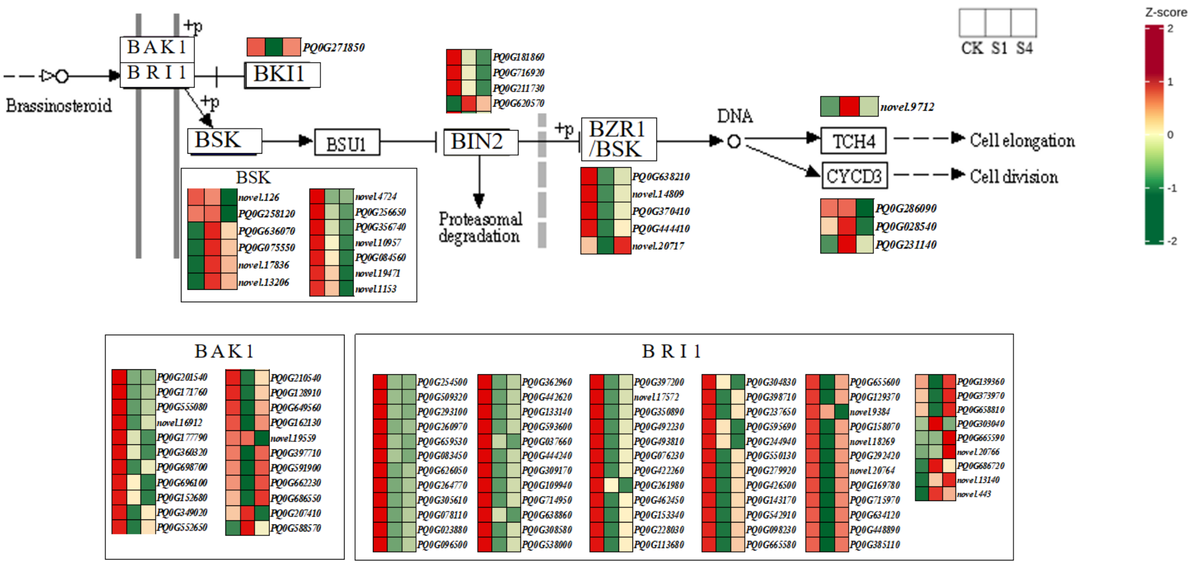

Figure S4 Expression profiles of structural genes involved in the BR signaling pathway in roots of American ginseng plants under foliar application of brassinolide. The grids shaded from blue to red represent logarithmic changes in DEGs. Brassinosteroid-insensitive 1 (BRI1), BRI1-associated kinase 1 (BAK1), Brassinosteroid-signaling kinase (BSK), BRI1 suppressor 1 (BSU1), BRI1 kinase inhibitor 1 (BKI1), Brassinosteroid-insensitive 2 (BIN2), Brassinazole-resistant 1 (BZR1), BRI1-ems-suppressor 1 (BES1), Xyloglucan endotransglycosylase/hydrolase 4 (TCH4), Cyclin D3 (CYCD3).

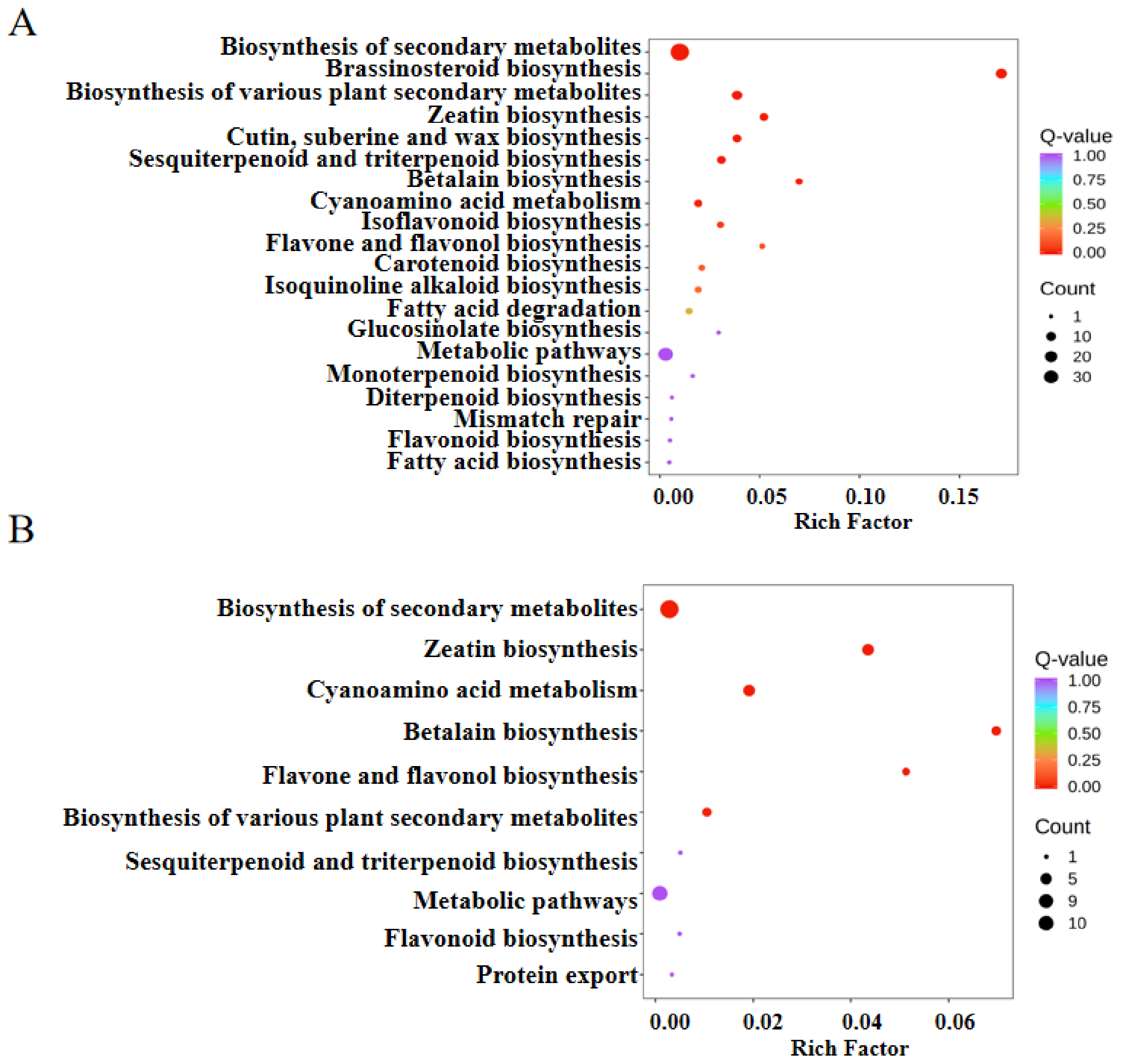

Figure S5 Kyoto Encyclopedia of Genes and Genomes (KEGG) enrichment analysis of differentially expressed genes in American ginseng roots after foliar application of BR to American ginseng plants. **(A)** KEGG enrichment analysis of 91 CYP450 and 30 UGT genes identified in the transcriptome. **(B)** KEGG enrichment analysis of 19 CYP450 genes, 3 UGT genes, and 184 transcription factors derived from three gene modules identified via weighted gene co-expression network analysis (WGCNA).

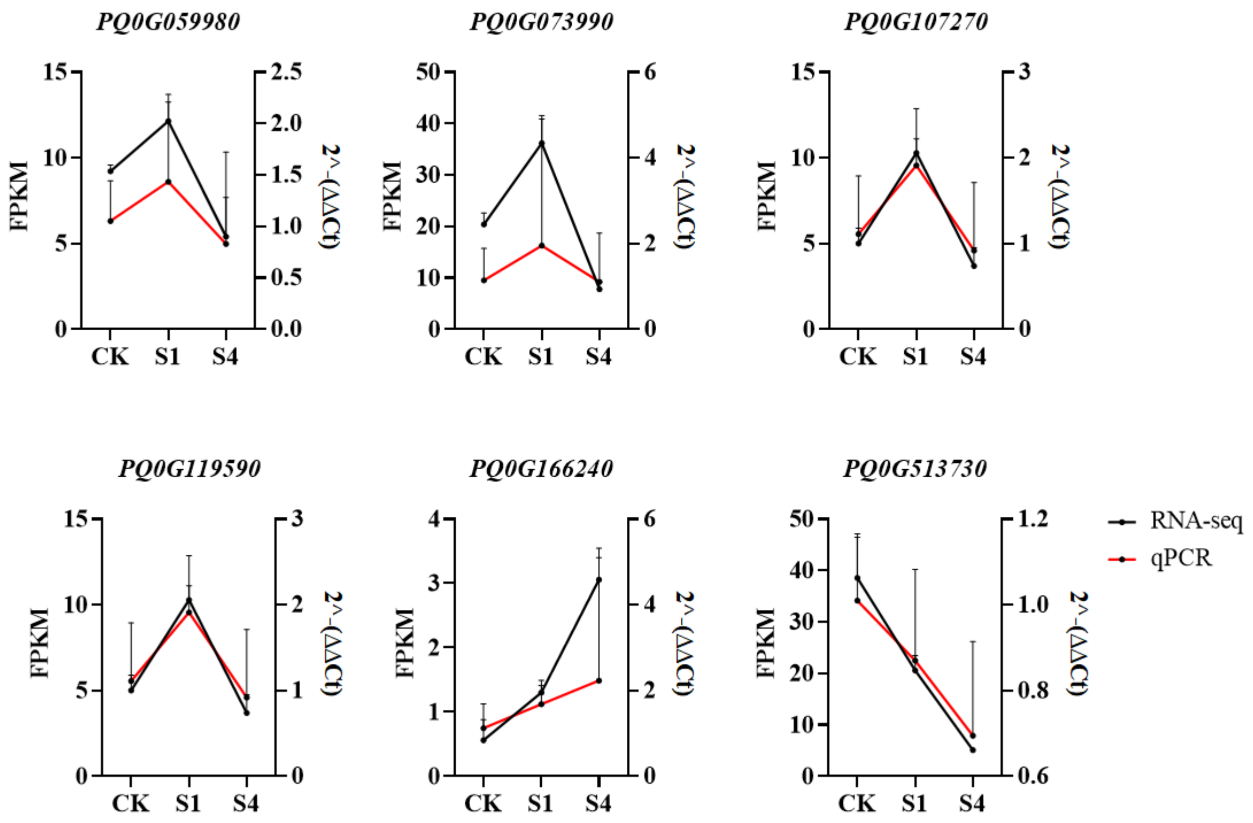

Figure S6 Validation of RNA-Seq expression levels with qPCR. Black and red lines represent relative gene expression levels observed in the RNA-Seq and qPCR respective datasets. RNA-seq data are Fragments per Kilobase per Million Reads (FPKM) normalized means ± SEM. Relative gene expression of qPCR data utilized using PqActin gene as reference gene and calculated with the 2(- Δ Δ Ct) algorithm.
